# Identification of Immune and Hypoxia Risk Classifier to Estimate Immune Microenvironment and Prognosis in Cervical Cancer

**DOI:** 10.1155/2022/6906380

**Published:** 2022-10-17

**Authors:** Yujing Shi, Qing Gao, Zeyuan Liu, Gefenqiang Shen, Xinchen Sun, Xiaoke Di

**Affiliations:** ^1^Department of Oncology, Jurong People's Hospital, Huayang Town, Jurong City, China; ^2^Department of Radiation Oncology, The First Affiliated Hospital of Nanjing Medical University, Nanjing, China; ^3^Department of Radiation Oncology, Nanjing Jiangning Hospital and the Affiliated Jiangning Hospital of Nanjing Medical University, Nanjing, China

## Abstract

**Purpose:**

Cervical cancer (CC) is one of the most common gynecologic neoplasms. Hypoxia is an essential trigger for activating immunosuppressive activity and initiating malignant tumors. However, the determination of the role of immunity and hypoxia on the clinical outcome of CC patients remains unclear.

**Methods:**

The CC independent cohort were collected from TCGA database. Consensus cluster analysis was employed to determine a molecular subtype based on immune and hypoxia gene sets. Cox relevant analyses were utilized to set up a risk classifier for prognosis assessment. The underlying pathways of classifier genes were detected by GSEA. Moreover, we conducted CIBERSORT algorithm to mirror the immune status of CC samples.

**Results:**

We observed two cluster related to immune and hypoxia status and found the significant difference in outcome of patients between the two clusters. A total of 251 candidate genes were extracted from the two clusters and enrolled into Cox relevant analyses. Then, seven hub genes (CCL20, CXCL2, ITGA5, PLOD2, PTGS2, TGFBI, and VEGFA) were selected to create an immune and hypoxia-based risk classifier (IHBRC). The IHBRC can precisely distinguish patient risk and estimate clinical outcomes. In addition, IHBRC was closely bound up with tumor associated pathways such as hypoxia, P53 signaling and TGF *β* signaling. IHBRC was also tightly associated with numerous types of immunocytes.

**Conclusion:**

This academic research revealed that IHBRC can be served as predictor for prognosis assessment and cancer treatment estimation in CC.

## 1. Introduction

Cervical cancer (CC) is the fourth most frequently diagnosed cancer and the second mortal cancer in female population, which poses a serious health threat to women globally [1]. According to the GLOBOCAN 2020 database, there were 604127 new cases and 341831 new deaths from CC, and the death rate is 12.4 versus 5.2 per 100,000 people in transitioning and in transited countries, respectively [2]. Etiologically, accumulating evidence has implied that infection with high-risk human papillomavirus (HPV) is the primary factor for CC [3]. Up to 90% of cases are driven by high-risk HPV strains including 16, 18, 31, 33, and 35, with other low-risk HPV types generally produce benign cervical lesions [4, 5]. Despite the promotion of HPV vaccine immunoprevention, many patients are diagnosed with advanced stage at their first diagnosis, making the exploration of early diagnosis biomarkers and effective prognostic model urgently needed [6, 7].

Recently, tumor microenvironment (TME) is causing general interest in various cancer settings. TME is composed of multiple cells residing in cancers, including immune cells, fibroblasts, endothelial cells, and mesenchymal cells [8]. These cells closely interact with each other and organize into distinct cellular communities [9]. Distinct immune cell response categories tumors into 3 types named “hot”, “altered”, and “cold” tumors [10]. Accumulating evidence has identified the immunotherapy as a promising intervention for cancer patients [11]. By reprograming the immunosuppressive state in the “cold tumor” into an activated one, the usage of immune checkpoint inhibitors as well as some cell-specific compounds has achieved exciting clinic outcome in multiple cancers [12, 13]. However, immunotherapy in CC remains largely unexplored.

Hypoxia is one of common characteristics of tumors and is closely related to tumor progression and poor prognosis [14, 15]. Cells respond to hypoxia environment by regulating various metabolism pathways, which subsequently causes deficient hypervascularization, enhanced tumor cell proliferation, and distant metastasis tendency [16–18]. Emerging evidence has validated the crosstalk between hypoxia and immunophenotype in tumors. For instance, HIF2*α* has been reported to exert its protective role in pancreatic ductal adenocarcinoma by improving immune responses [19]. Moreover, Zhang et al. once reported that hypoxia condition elevated the tumor cell resistance to cytotoxic T lymphocytes mediated lysis, which is dependent on the upregulation of HIF1*α* and PD-1 expression [20]. Taken together, it is reasonable to speculate that novel approaches targeting alleviating hypoxia condition could augment the current outcome for CC patients.

Most of the indicators proposed in previous studies to predict clinical outcomes of CC patients are limited to single genes, such as HPV, PTEN, and FHIT [21]. However, using only a single biomarker to assess prognosis is greatly partial, as the mechanisms affecting the development of CC are extremely complex. Currently, prognostic signature consisting of multiple genes has been proven to present independent prognostic ability by several reports, which has also attracted the attention of scholars in the field of oncology [22, 23]. Compared to the traditional TNM system, the prognostic model is capable of accurately predicting not only clinical outcomes but also the patient's immune status and treatment benefits.

The alteration of metabolic state and immunophenotype in tumors largely restrain the therapy response for CC patients, while the relevant study is still in very early stages. In our current research, we combined the immune-related genes (IRGs) and hypoxia-related genes (HRGs) to establish a prognostic signature with high accuracy for CC. In addition, immune cell infiltration analysis was performed in two risk groups of CC samples. Altogether, our exploration will help clarify the specific immune environment in different populations and provide new ideas and insights for the prevention and treatment of CC.

## 2. Methods

### 2.1. Data Acquisition

The TCGA-CSCC dataset containing gene expression and simple nucleotide variation was collected from TCGA website (https://cancergenome.nih.gov/). And the clinical data of TCGA-CSCC dataset was obtained from cBioPortal website (http://www.cbioportal.org/). Next, we combined the clinical traits of the two databases by patient ID. The exclusion criteria were set as follows: (1) histologic diagnosis is not CC; (2) samples without completed data for analysis; and (3) survival time of less than 30 days. Moreover, we extracted IRGs from ImmPort database (https://www.immport.org/shared/genelists/) and collected HRGs from MSigDB website (https://www.gsea-msigdb.org/gsea/msigdb/, Supplementary Table 1).

### 2.2. Gene Cluster Analysis

The consensus cluster algorithm was performed using the “ConsensusClusterPlus” package [24]. To determine the optimal cluster score, we assessed the Delta area and cumulative distribution function (CDF). Next, we compared clinical outcome discrepancies between different subtypes by survival analysis. We also utilized differential analysis to screen differentially expressed genes (DEGs) between different subtypes for subsequent analysis [25].

### 2.3. Development of a Risk Classifier

All CC samples were randomly divided into training set and validation set. The DEGs from cluster analysis were first subject to univariate analysis. Then, we enrolled the potential genes with prognostic value in multivariate analysis. Finally, we created immune- and hypoxia-based risk classifier (IHBRC) according to regression coefficients of each model factors. The risk equation is as follows: risk factor = ∑_*i*=1_^*n*^(*Coef*_*i*_ × Exp_*i*_); Coef_i_ is the coefficient of the classifier generated by Cox analyses, and Exp_i_ is the expression level of each model genes. The patients were divided into high- and low-risk groups according to the median risk score.

### 2.4. Survival Analysis

The differences in clinical outcome were detected between two risk groups by Kaplan-Meier analysis. ROC curves were plotted to test the reliability of IRBRC in assessing patients' outcomes. Univariate and multivariate analyses were applied to confirm the independent value of IHBRC in CC.

### 2.5. Gene Set Enrichment Analysis (GSEA)

The transcriptome data and risk groups information were enrolled into GSEA [26]. Next, we selected the hallmark, all v7. 5. symbols. Gmt in the MSigDB database as the reference gene set. The default weighted enrichment method was applied for 1000 enrichment analysis. The gene sets with *p* < 0.05 and *FDR* < 0.25 were considered as significantly enriched gene sets.

### 2.6. Immune Infiltration Analysis

CIBERSORT is a powerful algorithm proposed by Newman et al. to mirror the infiltration status of immunocytes [27]. Performing an immunocytes gene set including 547 genes, CIBERSORT was applied to determine 22 immunocyte types containing B cells (naive B cells and memory B cells), T cells (CD8 T cells, naïve CD4 T cells, resting memory CD4 T cells, activated memory CD4 T cells, follicular helper T cells), immunosuppressive cells (T cells regulatory (Tregs), M2 macrophages and eosinophils) as well as other cells (resting NK cells, activated NK cells, monocytes, macrophages, dendritic cells, mast cells, eosinophils, and plasma cells). To detect the TME of CC cases, we conducted correlation analysis to analyze the relationship between risk score and 22 immunocytes types.

### 2.7. Tumor Mutation Burden Analysis

We employed the mutation data of CC cases to compare the tumor mutation burden (TMB) in two subgroups. The TMB value was generated using following equation: *TMB* = (total mutation/total coverbased) × 10^6^.

### 2.8. Chemotherapy Drug Sensitivity Analysis

To estimate the predictive power of the IHBRC for chemotherapeutic drug efficacy, the half-maximal inhibitory concentration (IC50) was taken as an index to measure the drug sensitivity. The difference in the IC50 between two risk groups was compared by pRRophetic of R.

### 2.9. Identification of the Target miRNAs

To explore the target miRNAs of model genes, a prediction approach with starBase (http://starbase.sysu.edu.cn/) was conducted. The criteria for determination was set by five prediction programs.

## 3. Results

### 3.1. Characterization of Immune and Hypoxia Genes

**Figure 1:**
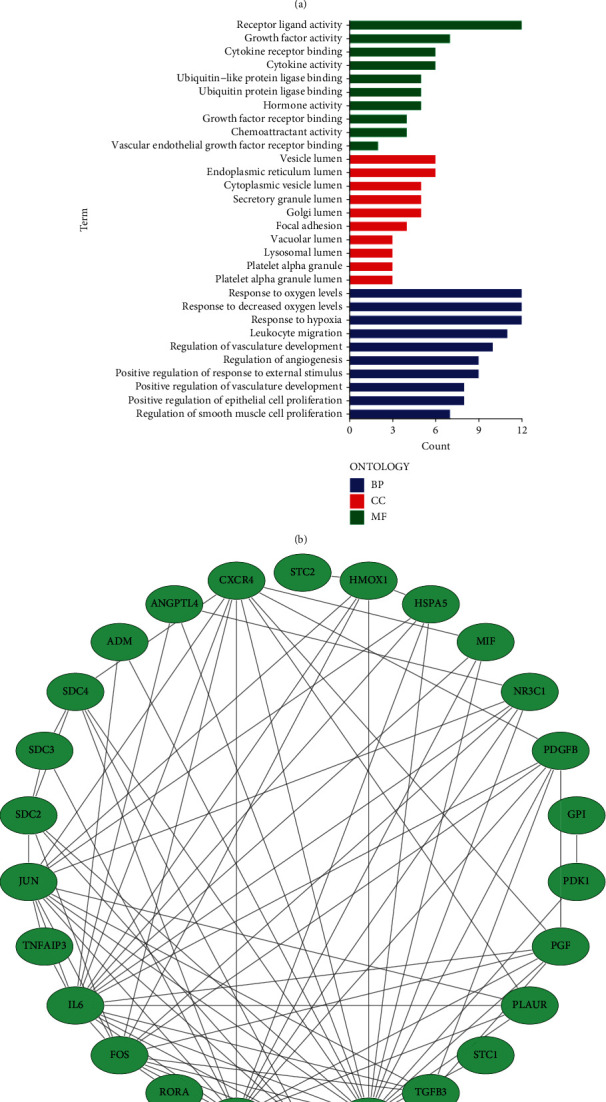
Characterization of immune and hypoxia genes. (a) The Venn plot of overlapped genes; (b) GO function enrichment analysis; (c) the PPI network of the overlapped genes.

To discover the hub genes which could regulate both immunity and hypoxia process, we screened 31 overlapped genes by intersection of IRGs and HRGs lists (Figure 1(a)). Then, we performed function analysis on these 31 genes and found that they were enriched in response to hypoxia, leukocyte migration, and regulation of angiogenesis (Figure 1(b)). Meanwhile, we created a PPI network to better clarify the interaction of 31 genes at protein level (Figure 1(c)).

### 3.2. Consensus Cluster Analysis

**Figure 2:**
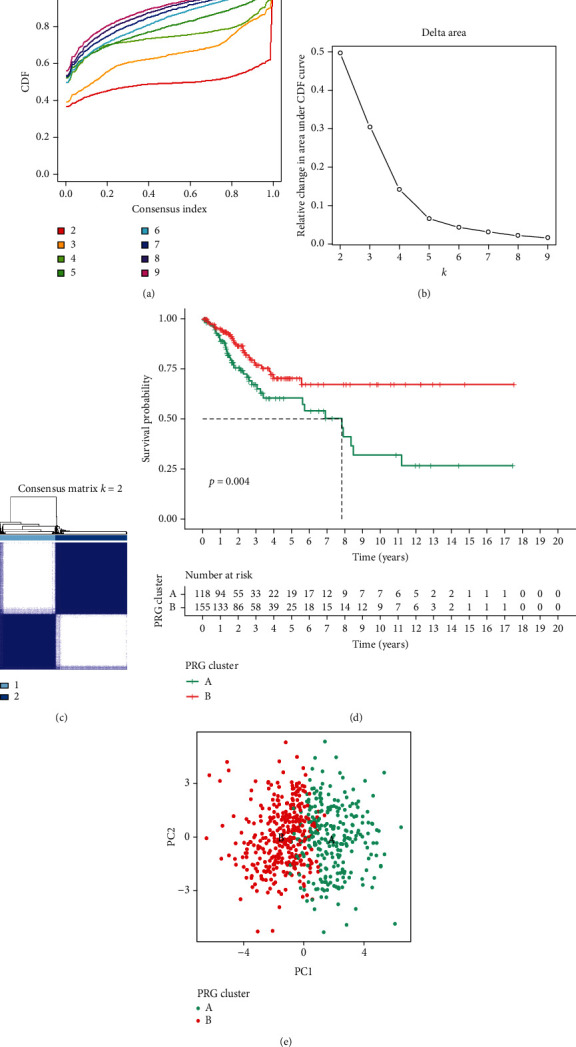
Consensus clustering determined a molecular subtype related to immune and hypoxia. (a) The CDF score of consensus index; (b) relative change of CDF curve; (c) consensus matrix for *k* = 2; (d) the Kaplan–Meier survival analysis; (e) principal component analysis of the two clusters.

A total of 31 hub genes were incorporated into cluster analysis. The results indicated that CDF value growth was flat when *k* = 2 and Delta area increased insignificantly at *k* > 3 (Figures 2(a) and 2(b)). The fractal matrix showed the favorable intergroup difference and intragroup association, suggesting these pivot genes could categorize all CC samples into two subtypes (Cluster 1 (*n* = 130) and Cluster 2 (*n* = 174)). Therefore, the clustering stability was best for *k* = 2 (Figure 2(c)). Survival analysis illustrated the significant difference in patient outcome between two clusters (Figure 2(d)). PCA analysis uncovered the favorable distinction between the two clusters (Figure 2(e)). Furthermore, 251 DEGs were collected from differential analysis between two clusters.

### 3.3. Development of a Risk Classifier

**Figure 3:**
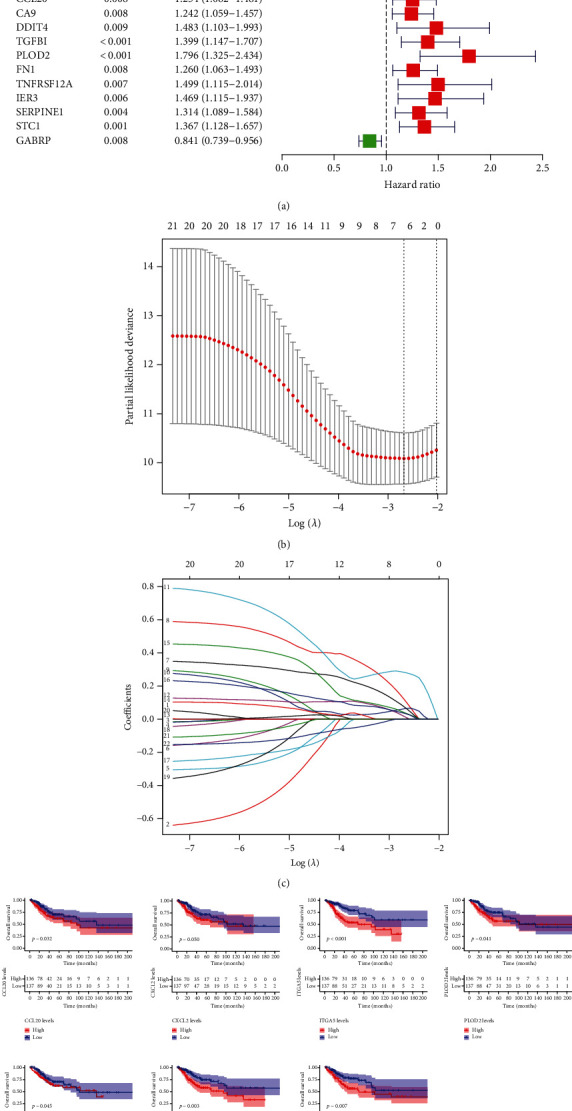
Construction of a risk classifier. (a) Univariate Cox regression analysis; (b–c) LASSO coefficients for risk classifier; (d) the survival analysis of classifier genes.

In the training set, we first determined 24 survival-associated indicators based on above 251 DEGs via univariate analysis (Figure 3(a)). Then, the candidate genes were enrolled into LASSO regression to remove the over fitting genes (Figures 3(b) and 3(c)). Finally, multivariate analysis was employed, and seven hub genes were selected to develop an IHBRC (Table 1): risk score = (0.0131 × CCL20) + (0.0638 × CXCL2) + (0.2812 × ITGA5) + (0.0340 × PLOD2) + (0.0697 × PTGS2) + (0.0374 × TGFBI) + (0.1113 × VEGFA). In addition, Figure 3(d) demonstrated the prognostic power of seven hub predictors.

**Figure 4:**
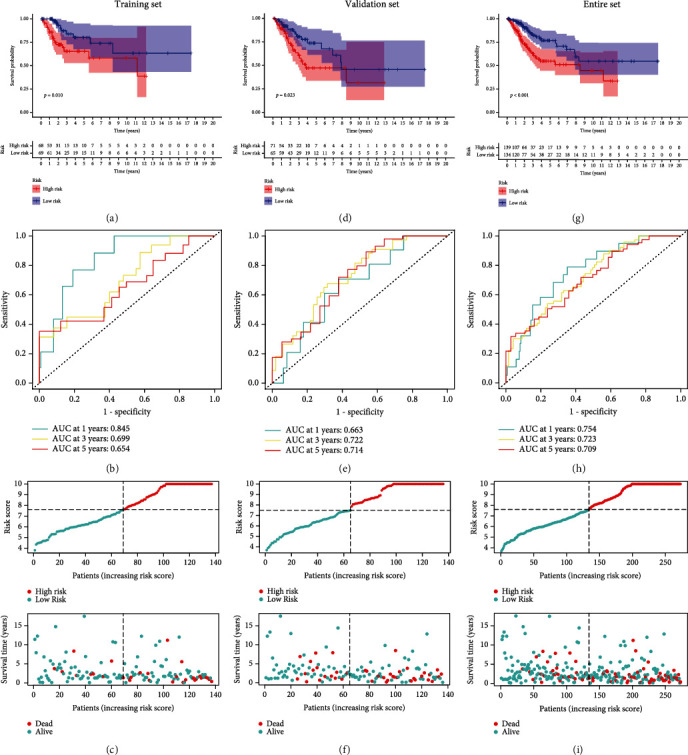
Predictive value of the classifier. (a) Survival curves of prognostic difference between two risk groups in the training set; (b) ROC curve of the assessment reliability of the classifier in the training set; (c) the distribution of risk score and survival status in the training set. (d–f) and (g–i) the testing set and the entire set were used to confirm the predictive value of the classifier.

As suggested by Figure 4(a), high-risk group presented a dismal prognosis benefit in the training set. The AUC values of 1-, 3-, and 5-year survival were 0.845, 0.699, and 0.654, respectively (Figure 4(b)). We measured the survival outcome of patients in both groups and found that patients' outcomes were dismal as the risk score elevated (Figure 4(c)). Meanwhile, we confirmed the performance of IHBRC in the validation and the entire cohorts using the same analysis described above and obtained the same results for the trend (Figures 4(d)–4(i)).

### 3.4. Independent Prognostic Analysis

**Figure 5:**
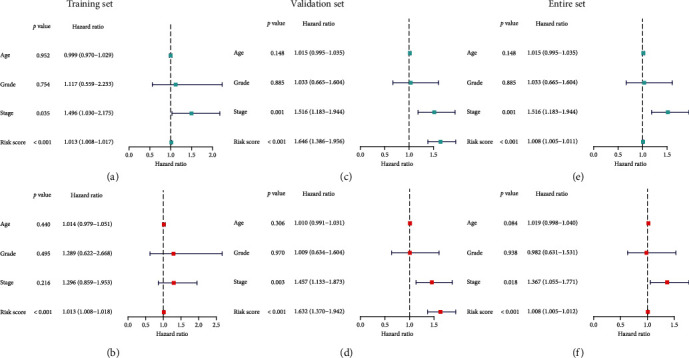
Independent prognosis analysis of the classifier. (a–c) Univariate Cox regression analysis; (d–f) multivariate Cox regression analysis.

To examine the independent value of IHBRC in terms of survival of CC cases, univariate and multivariate analyses were employed. In the training set, univariate analysis demonstrated that low risk score was remarkably correlated with favorable prognosis (Figure 5(a)). Furthermore, multivariate analysis still revealed that low risk score was independently associated with favorable outcome of CC patients (Figure 5(b)), which could serve as an independent prognostic factor for glioma. These were confirmed by the test and the entire sets (Figures 5(c)–5(f)).

### 3.5. GSEA Enrichment Analysis

**Figure 6:**
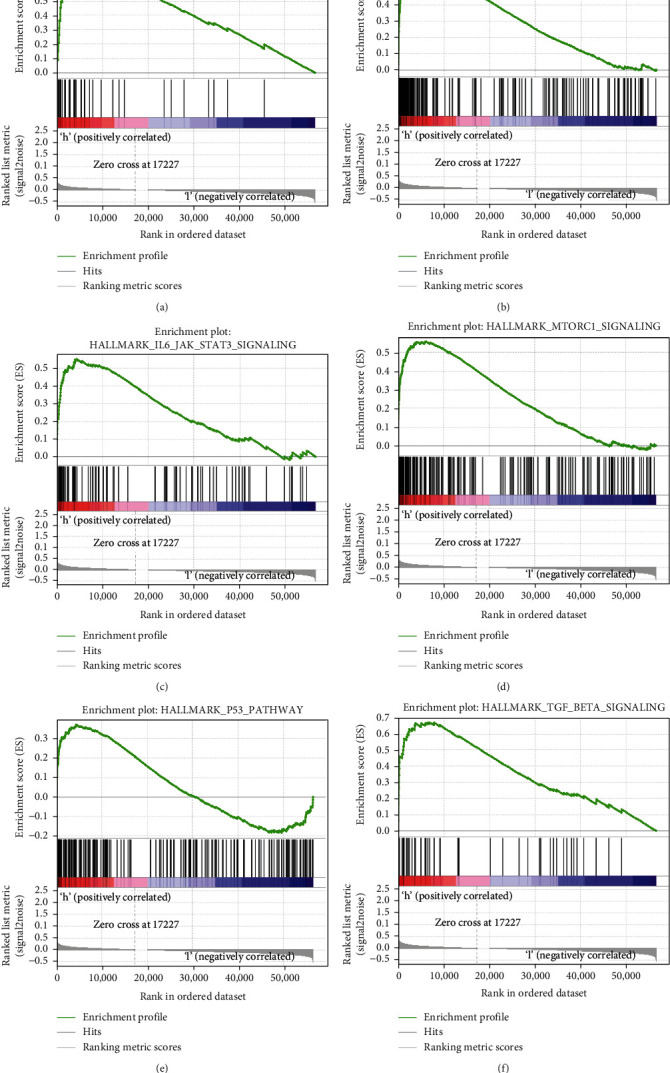
Gene set enrichment analysis. (a) Angiogenesis; (b) hypoxia; (c) IL6/JAK/STAT3 signaling; (d) MTORC1 signaling; (e) P53 signaling; (f) TGF *β* signaling.

To explore the distinction in molecular pathways between the two groups, we applied GSEA based on hallmarks gene sets. The results disclosed that hallmarks including angiogenesis, hypoxia, IL6/JAK/STAT3 signaling, MTORC1 signaling, P53 signaling, and TGF *β* signaling were markedly enriched in high-risk group (Figure 6).

### 3.6. Immune Infiltration Analysis

**Figure 7:**
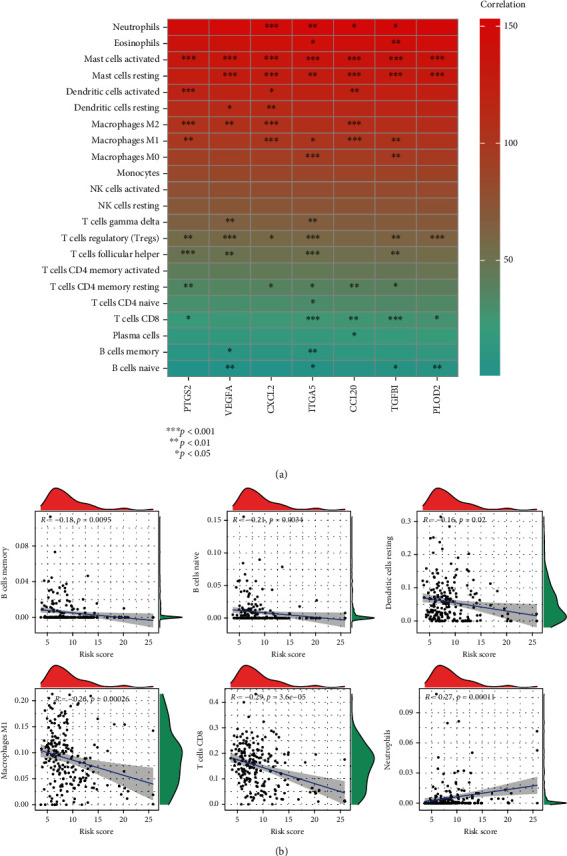
Immune infiltration analysis. (a) The relationship between seven model biomarkers and immunocytes; (b) correlation analysis of risk score and immunocytes (memory B cells, naïve B cells, resting dendritic cells, macrophages M1, CD8 T cells, and neutrophils).

In order to mirror the immune status of two groups, we estimated enrichment value of different immunocytes.  Figure 7(a) illustrated the relationship between seven model biomarkers and immunocytes. As shown in Figure 7(b), risk score was negatively correlated with the infiltration level of memory B cells, naïve B cells, resting dendritic cells, and macrophages M1 and CD8 T cells, while neutrophils were activated in IHBRC-high cohort.

### 3.7. Immune Checkpoints Analysis for Risk Classifier

**Figure 8:**
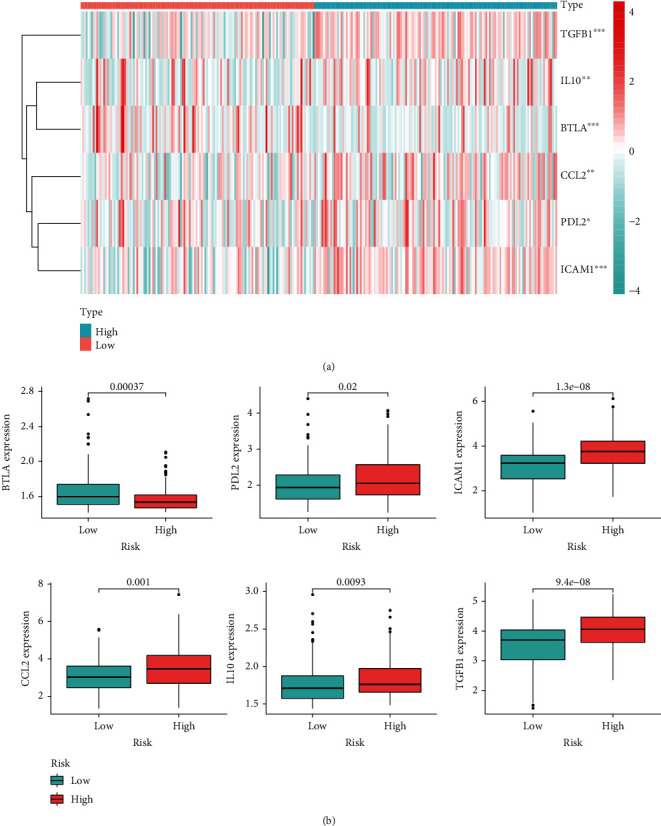
Correlation analysis of immune checkpoints and risk score. (a) Heatmap displaying expression patterns of immune checkpoints between two groups; (b) box line diagram showing the expression differences of six immune checkpoints between two groups.

Subsequently, we detected the relationship between signature and the expression of immune checkpoints.  Figure 8(a) revealed six immune checkpoints that were greatly differentially expressed in the two risk groups. As suggested by Figure 8(b), BTLA was significantly downregulated in the high-risk group, while PDL2, ICAM1, CCL2, IL10, and TGFB1 were markedly enriched in the high-risk group, indicating that patients with high risk are likely to be immunosuppressive status.

### 3.8. Analysis of Immunotherapy and Chemotherapy Response

**Figure 9:**
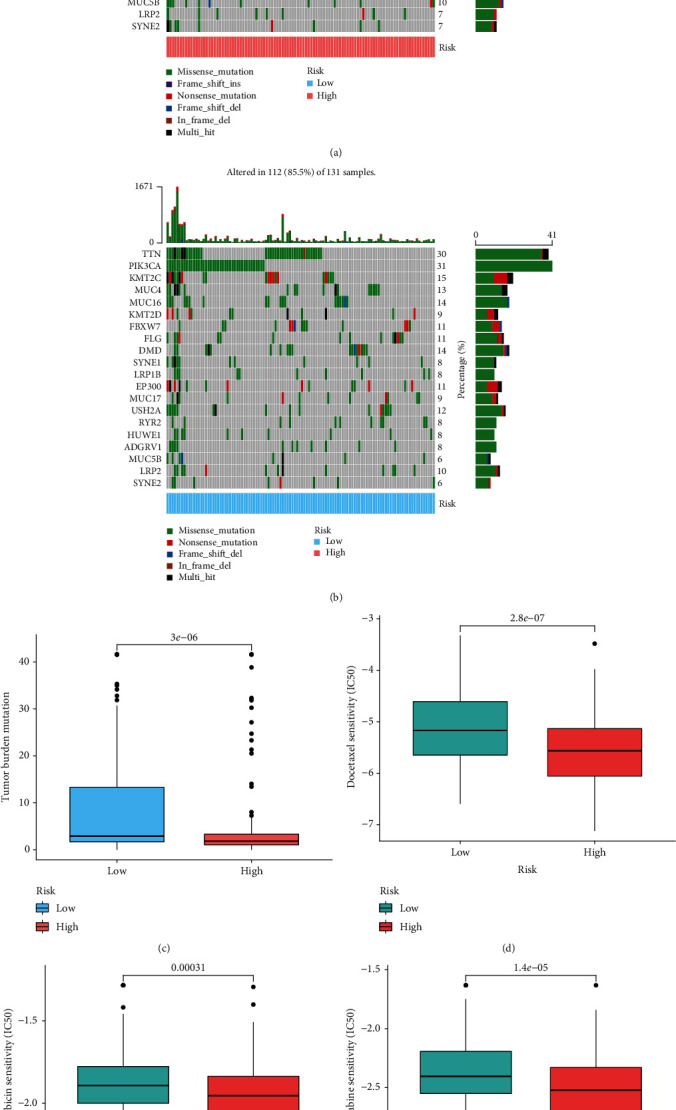
Analysis of immunotherapy and chemotherapy response. (a–b) The top 20 mutated genes in the two groups; (c) the TMB in the two groups; (d–f) chemotherapeutic response in the two groups.

Waterfall diagrams indicated the mutational differences in the 20 genes between the two groups. We observed that the IHBRC-high cohort had a higher PIK3CA mutation rate than the IHBRC-low group (31 vs. 20%), (Figures 9(a) and 9(b)). Given the importance of TMB in evaluating immunotherapy response for patients with CC, we observed IHBRC-high group had lower TMB value (Figure 9(c)). In addition, high risk score was correlated with a lower IC50 of docetaxel, doxorubicin, and gemcitabine (*p* < 0.05), suggesting that the IHBRC served as a favorable indicator for chemosensitivity (Figures 9(d)–9(f)).

### 3.9. Construction of IHBRC-Related Regulatory Network

**Figure 10:**
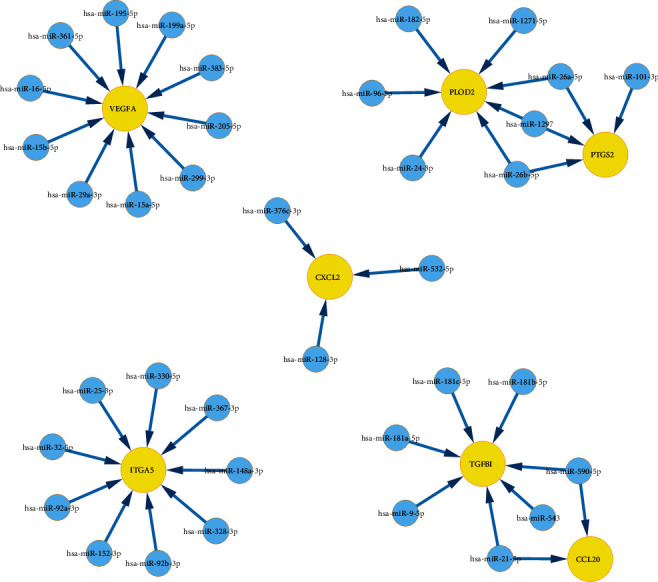
Construction of IHBRC-related regulatory network.

The reciprocal regulation of mRNA and miRNA is closely bound up with tumor development. Based on the starbase online tool, we identified the target miRNAs of seven model genes with high relevance scores (Figure 10). Moreover, miRNA set enrichment analysis was performed to explore the function of the target miRNAs by TAM 2.0 tool. The results showed these miRNAs were mainly involved in cell aging, apoptosis, immune response, inflammation, and regulation of Stem Cell (Supplementary Table 2).

## 4. Discussion

Antitumor effects of immune cells could be largely influenced by TME, including intercellular crosstalk between different cell types, chemokines concentrations, and metabolism environment, thus it is crucial to establish a comprehensive understanding on the genetic and population characteristics of TME. In our study, we categorize CC patients into two distinct clusters, in which they have totally differed prognosis, based on the expression level of immune- and hypoxia-related genes. Our proposed classifier is a favorable biomarker to assess the prognosis of CC cases. Meanwhile, the classifier can serve as an indicator for predicting immune infiltration levels, TMB value and chemotherapy response, providing a novel insight for future research and clinical practice.

A total of hub seven genes (CCL20, CXCL2, ITGA5, PLOD2, PTGS2, TGFBI, and VEGFA) were identified as risky indicators in our prognostic model, and the involvement of some genes in CC has been reported before. PTGS2, also named COX-2, is a crucial target to prevent progression in various cancer types [28–30]. Early in 2004, Kulkarni et al. reported that the COX-2 expression was elevated in CC samples compared to normal cervical tissue. A number of signaling including EGF and nuclear factor *κ*B (NF-*κ*B) pathway has been validated to mediate COX-2 expression in CC [31, 32]. Moreover, the usage of COX-2 selective inhibitors selectively enhances radio responsiveness in CC cell line under both normoxic and hypoxic conditions [33]. VEGFA is considered to play a crucial role in physiological and pathological angiogenesis [34]. In stimulation of VEGFA, endothelial cells proliferate and migrate to form new vessels [35]. The cross talk between VEGF signaling and immune response has been recently demonstrated. Briefly, VEGFA contributes to the polarization of macrophages into an M2 immunosuppressive phenotype [36–38]. In turn, these immunosuppressive cells can further produce proangiogenic factors including VEGFA and MMP9 [39]. The role of CXCL2 in CC has been intensively reported before. Zhang et al. once revealed that CXCL2 may promote tumor proliferation and metastasis induced by the overexpression of A-kinase-interacting protein 1 (AKIP1) in CC [40]. In agreement with our result, Yang et al. recently indicated that the expression level CXCL2 is strongly associated with lymph node metastasis and prognosis in CC patients [41]. Four other genes including ITGA5, CCL20, TGFBI, and PLOD2 were previously studied in various malignancies, while their involvement in CC remains largely unexplored, and more basic researches are needed to reveal their biological function in CC [42–44].

As an endogenous noncoding RNA, miRNA could regulate 30% of protein-coding genes in human cells. Numerous studies have reported that miRNA is an upstream regulator of tumor-associated genes and engages in regulating biological processes such as proliferation and migration of cancer cells [45]. Our results revealed that hsa-miR-26a-5p, has-miR-26b-5p, hsa-miR-1297, hsa-miR-590-5p, and hsa-miR-21-5p were shared modulators of model genes. In cervical cancer, miR-590-5p was proven to facilitate tumor viability by inhibiting CHL1 [46]. Also, miR-590-5p could boost the malignant behaviors of liver cancer by interacting with FOXO1 [47]. Gu et al. disclosed that DUXAP8 could boost cells growth and angiogenesis by targeting miR-1297 in CC [48]. Moreover, miR-21-5p also serves as an important factor regulating the effect of HAND2-AS1 on CC [49].

Molecular signaling was further analyzed in our research to unveil the mechanism underlying CC progression. In general, defective vasculatures and overweighing demands of oxygen contribute to the hypoxia environment in solid tumors [50]. HIFs induced by the hypoxic microenvironment play a central part in several aspects of tumor formation, especially in the regulation of tumor angiogenesis. HIF has a bidirectional regulatory effect on tumor angiogenesis. In vitro studies revealed that when HIF-1*α* activity was inhibited, it had different effects on the expression of proangiogenic factors. VEGF, angiogenin, and TGF*β*-1 expressions were diminished, while IL-6 and MCP-1 were significantly increased. In vivo tests showed that RNA inhibition of HIF-1*α* also showed a decrease in VEGF expression and an increase in IL-8 expression. Consequently, when HIF-*α* is inhibited, one proangiogenic factor may be increased when another proangiogenic factor is inhibited, and as a result, there may still be an actual increase in tumor vascularization [51, 52].

As a result, ATP production shifts from oxidative phosphorylation to glycolysis, and the acidic microenvironments subsequently confer the alterations of gene expression and activation of multiple molecular pathways, accelerating the cancer progression [53, 54]. The genetic alternations of mTOR protein have a significant role in tumorigenesis [55, 56]. A number of molecules are involved in the modulation of mTOR signaling, and specific inhibitors show a good performance in prevention and treatment of various tumors including oral cancer, ovarian cancer, and lung carcinoma [57–59]. TP53, which encodes a sequence-specific DNA-binding transcription factor, is one of the most frequently mutated genes in cancers [60]. Studies show that depletion of TP53 can remarkably increase the incidence of carcinogen-induced carcinogenesis and accelerate the tumor growth and invasiveness [61]. TGFB is a critical regulator of numerous biological processes in both normal and cancer cells [62]. Timmins and Ringshausen recently reviewed that in B-cell malignancies, targeting the TGFB axis, should be considered a promising approach in the context of immunotherapy [63]. The IL-6/STAT3 pathway is a classic signaling that can induce enhanced EMT process in cancers [64]. You et al. revealed the function role of IL-6/STAT3 pathway in promoting the malignant progression in oral squamous cell carcinoma patients, and further research is urgently needed to establish a more applicable therapeutic strategy targeting STAT3 pathway [65].

Of note, the immune landscape results validated that the infiltration level of M1-like macrophage and antitumor CD8+ T cells is significantly low in high-risk group, which is associated with poor clinical outcome. It has been indicated that M1-like macrophage serve as a protective factor in tumor microenvironment by promoting antitumor response [66, 67]. For instance, a recent study pointed out that irradiation in CC can bring a subtype shift from M2-like to the M1-like phenotype and eventually lead to an enhanced antitumor immune status [68]. It is well established that CD8+ T cells play key roles in the elimination of HPV in CC [69]. Previous studies have uncovered the higher ratios of CD8+ to CD4+ T cells being closely related to improved survival [70]. On the contrary, the infiltration of neutrophils is proven to be positively correlated with survival of CC patients in our model, which is consistent with the common view that neutrophils are regarded as the most important leukocytes involving in first line defense to tissue damage [71–73]. Compared to the classic discipline to divide tumor immunophenotype into three subtypes (hot, altered, and cold), our finding compared the immune cell infiltration in high- and low-risk populations, may provide a more accurate model to guide the cellular based immunotherapy in CC.

Considerable research has suggested that docetaxel, doxorubicin, and gemcitabine can be the major chemotherapy drugs to control CC [74–76]. Exploring the relationship between risk and chemotherapy sensitivity by our model, we observed that high-risk patients had a higher sensitivity to the above drugs, which provides a favorable reference for the chemotherapy strategy of cases with CC.

Although our model was confirmed to possess promising potential for clinical application in CC, our project has some shortcomings. The clinical cohort in our study was drawn from the TCGA database of samples. We still need external datasets to validate our model. In addition, our research was mainly based on bioinformatics analyses, the expression pattern and underlying mechanisms of the model be detected with *in vivo* and *in vitro* experiments.

In summary, we developed a favorable risk classifier according to immune and hypoxia molecular subtypes. Our proposed risk classifier can be served as predictor for prognosis assessment and cancer treatment estimation in CC.

## Figures and Tables

**Table 1:** Multivariate analysis of the seven model genes in CC.

Gene	Coefficient	*P* value
CCL20	0.0131	0.007
CXCL2	0.0638	0.001
ITGA5	0.2812	0.001
PLOD2	0.0340	0.001
PTGS2	0.0697	0.008
TGFBI	0.0374	0.001
VEGFA	0.1113	0.001

## Data Availability

The public datasets to support the results of this subject can be gained from TCGA (https://portal.gdc.cancer.gov/).

## Supplementary Materials

Supplementary 1 Supplementary Table 1: the list of hypoxia-related genes.

Supplementary 2 Supplementary Table 2: potential function of the target miRNAs.
