# Supplementary material for: Identification of Immune and Hypoxia Risk Classifier to Estimate Immune Microenvironment and Prognosis in Cervical Cancer
Source: J Oncol. 2022 Oct 17;2022:6906380. doi: 10.1155/2022/6906380 (PMC9593224; doi:10.1155/2022/6906380)
Supplement: Supplementary 1 — Supplementary Table 1: the list of hypoxia-related genes. [file 6906380.f1.zip › Supplementary Table 1 (1).pdf]

Supplementary Table 1: The list of hypoxia-related genes.

Original Member	NCBI (Entrez) Gene Id	Gene Symbol
ADM	133	ADM
ADORA2B	136	ADORA2B
AK4	205	AK4
AKAP12	9590	AKAP12
ALDOA	226	ALDOA
ALDOB	229	ALDOB
ALDOC	230	ALDOC
AMPD3	272	AMPD3
ANGPTL4	51129	ANGPTL4
ANKZF1	55139	ANKZF1
ANXA2	302	ANXA2
ATF3	467	ATF3
ATP7A	538	ATP7A
B3GALT6	126792	B3GALT6
B4GALNT2	124872	B4GALNT2
BCAN	63827	BCAN
BCL2	596	BCL2
BGN	633	BGN
BHLHE40	8553	BHLHE40
BNIP3L	665	BNIP3L
BRS3	680	BRS3
BTG1	694	BTG1
CA12	771	CA12
CASP6	839	CASP6
CAV1	857	CAV1
CCNG2	901	CCNG2
CCRN4L	25819	NOCT
CDKN1A	1026	CDKN1A
CDKN1B	1027	CDKN1B
CDKN1C	1028	CDKN1C
CHST2	9435	CHST2
CHST3	9469	CHST3
CITED2	10370	CITED2
COL5A1	1289	COL5A1
CP	1356	CP
CSRP2	1466	CSRP2
CTGF	1490	CCN2
CXCR4	7852	CXCR4
CXCR7	57007	ACKR3
CYR61	3491	CCN1

DCN	1634	DCN
DDIT3	1649	DDIT3
DDIT4	54541	DDIT4
DPYSL4	10570	DPYSL4
DTNA	1837	DTNA
DUSP1	1843	DUSP1
EDN2	1907	EDN2
EFNA1	1942	EFNA1
EFNA3	1944	EFNA3
EGFR	1956	EGFR
ENO1	2023	ENO1
ENO2	2026	ENO2
ENO3	2027	ENO3
ERO1L	30001	ERO1A
ERRFI1	54206	ERRFI1
ETS1	2113	ETS1
EXT1	2131	EXT1
F3	2152	F3
FAM162A	26355	FAM162A
FBP1	2203	FBP1
FOS	2353	FOS
FOSL2	2355	FOSL2
FOXO3	2309	FOXO3
GAA	2548	GAA
GALK1	2584	GALK1
GAPDH	2597	GAPDH
GAPDHS	26330	GAPDHS
GBE1	2632	GBE1
GCK	2645	GCK
GCNT2	2651	GCNT2
GLRX	2745	GLRX
GPC1	2817	GPC1
GPC3	2719	GPC3
GPC4	2239	GPC4
GPI	2821	GPI
GRHPR	9380	GRHPR
GYS1	2997	GYS1
HAS1	3036	HAS1
HDLBP	3069	HDLBP
HEXA	3073	HEXA
HK1	3098	HK1
HK2	3099	HK2
HMOX1	3162	HMOX1

HOXB9	3219	HOXB9
HS3ST1	9957	HS3ST1
HSPA5	3309	HSPA5
IDS	3423	IDS
IER3	8870	IER3
IGFBP1	3484	IGFBP1
IGFBP3	3486	IGFBP3
IL6	3569	IL6
ILVBL	10994	ILVBL
INHA	3623	INHA
IRS2	8660	IRS2
ISG20	3669	ISG20
JMJD6	23210	JMJD6
JUN	3725	JUN
KDELR3	11015	KDELR3
KDM3A	55818	KDM3A
KIF5A	3798	KIF5A
KLF6	1316	KLF6
KLF7	8609	KLF7
KLHL24	54800	KLHL24
LALBA	3906	LALBA
LARGE	9215	LARGE1
LDHA	3939	LDHA
LDHC	3948	LDHC
LOX	4015	LOX
LXN	56925	LXN
MAFF	23764	MAFF
MAP3K1	4214	MAP3K1
MIF	4282	MIF
MT1E	4493	MT1E
MT2A	4502	MT2A
MXI1	4601	MXI1
MYH9	4627	MYH9
NAGK	55577	NAGK
NCAN	1463	NCAN
NDRG1	10397	NDRG1
NDST1	3340	NDST1
NDST2	8509	NDST2
NEDD4L	23327	NEDD4L
NFIL3	4783	NFIL3
NR3C1	2908	NR3C1
P4HA1	5033	P4HA1
P4HA2	8974	P4HA2

PAM	5066	PAM
PCK1	5105	PCK1
PDGFB	5155	PDGFB
PDK1	5163	PDK1
PDK3	5165	PDK3
PFKFB3	5209	PFKFB3
PFKL	5211	PFKL
PFKP	5214	PFKP
PGAM2	5224	PGAM2
PGF	5228	PGF
PGK1	5230	PGK1
PGM1	5236	PGM1
PGM2	55276	PGM2
PHKG1	5260	PHKG1
PIM1	5292	PIM1
PKLR	5313	PKLR
PKP1	5317	PKP1
PLAC8	51316	PLAC8
PLAUR	5329	PLAUR
PLIN2	123	PLIN2
PNRC1	10957	PNRC1
PPARGC1A	10891	PPARGC1A
PPFIA4	8497	PPFIA4
PPP1R15A	23645	PPP1R15A
PPP1R3C	5507	PPP1R3C
PRDX5	25824	PRDX5
PRKCA	5578	PRKCA
PRKCDBP	112464	CAVIN3
PTRF	284119	CAVIN1
PYGM	5837	PYGM
RBPJ	3516	RBPJ
RORA	6095	RORA
RRAGD	58528	RRAGD
S100A4	6275	S100A4
SAP30	8819	SAP30
SCARB1	949	SCARB1
SDC2	6383	SDC2
SDC3	9672	SDC3
SDC4	6385	SDC4
SELENBP1	8991	SELENBP1
SERPINE1	5054	SERPINE1
SIAH2	6478	SIAH2
SLC25A1	6576	SLC25A1

SLC2A1	6513	SLC2A1
SLC2A3	6515	SLC2A3
SLC2A5	6518	SLC2A5
SLC37A4	2542	SLC37A4
SLC6A6	6533	SLC6A6
SRPX	8406	SRPX
STBD1	8987	STBD1
STC1	6781	STC1
STC2	8614	STC2
SULT2B1	6820	SULT2B1
TES	26136	TES
TGFB3	7043	TGFB3
TGFBI	7045	TGFBI
TGM2	7052	TGM2
TIPARP	25976	TIPARP
TKTL1	8277	TKTL1
TMEM45A	55076	TMEM45A
TNFAIP3	7128	TNFAIP3
TPBG	7162	TPBG
TPD52	7163	TPD52
TPI1	7167	TPI1
TPST2	8459	TPST2
UGP2	7360	UGP2
VEGFA	7422	VEGFA
VHL	7428	VHL
VLDLR	7436	VLDLR
WISP2	8839	CCN5
WSB1	26118	WSB1
XPNPEP1	7511	XPNPEP1
ZFP36	7538	ZFP36
ZNF292	23036	ZNF292
