# Supplementary material for: Identification of Immune and Hypoxia Risk Classifier to Estimate Immune Microenvironment and Prognosis in Cervical Cancer
Source: J Oncol. 2022 Oct 17;2022:6906380. doi: 10.1155/2022/6906380 (PMC9593224; doi:10.1155/2022/6906380)
Supplement: Supplementary 1 — Supplementary Table 1: the list of hypoxia-related genes. [file 6906380.f1.zip › Supplementary Table 1.docx]

Supplementary Table 1: The list of hypoxia-related genes.

| Original Member | NCBI (Entrez) Gene Id | Gene Symbol |
| --- | --- | --- |
| ADM | 133 | ADM |
| ADORA2B | 136 | ADORA2B |
| AK4 | 205 | AK4 |
| AKAP12 | 9590 | AKAP12 |
| ALDOA | 226 | ALDOA |
| ALDOB | 229 | ALDOB |
| ALDOC | 230 | ALDOC |
| AMPD3 | 272 | AMPD3 |
| ANGPTL4 | 51129 | ANGPTL4 |
| ANKZF1 | 55139 | ANKZF1 |
| ANXA2 | 302 | ANXA2 |
| ATF3 | 467 | ATF3 |
| ATP7A | 538 | ATP7A |
| B3GALT6 | 126792 | B3GALT6 |
| B4GALNT2 | 124872 | B4GALNT2 |
| BCAN | 63827 | BCAN |
| BCL2 | 596 | BCL2 |
| BGN | 633 | BGN |
| BHLHE40 | 8553 | BHLHE40 |
| BNIP3L | 665 | BNIP3L |
| BRS3 | 680 | BRS3 |
| BTG1 | 694 | BTG1 |
| CA12 | 771 | CA12 |
| CASP6 | 839 | CASP6 |
| CAV1 | 857 | CAV1 |
| CCNG2 | 901 | CCNG2 |
| CCRN4L | 25819 | NOCT |
| CDKN1A | 1026 | CDKN1A |
| CDKN1B | 1027 | CDKN1B |
| CDKN1C | 1028 | CDKN1C |
| CHST2 | 9435 | CHST2 |
| CHST3 | 9469 | CHST3 |
| CITED2 | 10370 | CITED2 |
| COL5A1 | 1289 | COL5A1 |
| CP | 1356 | CP |
| CSRP2 | 1466 | CSRP2 |
| CTGF | 1490 | CCN2 |
| CXCR4 | 7852 | CXCR4 |
| CXCR7 | 57007 | ACKR3 |
| CYR61 | 3491 | CCN1 |
| DCN | 1634 | DCN |
| DDIT3 | 1649 | DDIT3 |
| DDIT4 | 54541 | DDIT4 |
| DPYSL4 | 10570 | DPYSL4 |
| DTNA | 1837 | DTNA |
| DUSP1 | 1843 | DUSP1 |
| EDN2 | 1907 | EDN2 |
| EFNA1 | 1942 | EFNA1 |
| EFNA3 | 1944 | EFNA3 |
| EGFR | 1956 | EGFR |
| ENO1 | 2023 | ENO1 |
| ENO2 | 2026 | ENO2 |
| ENO3 | 2027 | ENO3 |
| ERO1L | 30001 | ERO1A |
| ERRFI1 | 54206 | ERRFI1 |
| ETS1 | 2113 | ETS1 |
| EXT1 | 2131 | EXT1 |
| F3 | 2152 | F3 |
| FAM162A | 26355 | FAM162A |
| FBP1 | 2203 | FBP1 |
| FOS | 2353 | FOS |
| FOSL2 | 2355 | FOSL2 |
| FOXO3 | 2309 | FOXO3 |
| GAA | 2548 | GAA |
| GALK1 | 2584 | GALK1 |
| GAPDH | 2597 | GAPDH |
| GAPDHS | 26330 | GAPDHS |
| GBE1 | 2632 | GBE1 |
| GCK | 2645 | GCK |
| GCNT2 | 2651 | GCNT2 |
| GLRX | 2745 | GLRX |
| GPC1 | 2817 | GPC1 |
| GPC3 | 2719 | GPC3 |
| GPC4 | 2239 | GPC4 |
| GPI | 2821 | GPI |
| GRHPR | 9380 | GRHPR |
| GYS1 | 2997 | GYS1 |
| HAS1 | 3036 | HAS1 |
| HDLBP | 3069 | HDLBP |
| HEXA | 3073 | HEXA |
| HK1 | 3098 | HK1 |
| HK2 | 3099 | HK2 |
| HMOX1 | 3162 | HMOX1 |
| HOXB9 | 3219 | HOXB9 |
| HS3ST1 | 9957 | HS3ST1 |
| HSPA5 | 3309 | HSPA5 |
| IDS | 3423 | IDS |
| IER3 | 8870 | IER3 |
| IGFBP1 | 3484 | IGFBP1 |
| IGFBP3 | 3486 | IGFBP3 |
| IL6 | 3569 | IL6 |
| ILVBL | 10994 | ILVBL |
| INHA | 3623 | INHA |
| IRS2 | 8660 | IRS2 |
| ISG20 | 3669 | ISG20 |
| JMJD6 | 23210 | JMJD6 |
| JUN | 3725 | JUN |
| KDELR3 | 11015 | KDELR3 |
| KDM3A | 55818 | KDM3A |
| KIF5A | 3798 | KIF5A |
| KLF6 | 1316 | KLF6 |
| KLF7 | 8609 | KLF7 |
| KLHL24 | 54800 | KLHL24 |
| LALBA | 3906 | LALBA |
| LARGE | 9215 | LARGE1 |
| LDHA | 3939 | LDHA |
| LDHC | 3948 | LDHC |
| LOX | 4015 | LOX |
| LXN | 56925 | LXN |
| MAFF | 23764 | MAFF |
| MAP3K1 | 4214 | MAP3K1 |
| MIF | 4282 | MIF |
| MT1E | 4493 | MT1E |
| MT2A | 4502 | MT2A |
| MXI1 | 4601 | MXI1 |
| MYH9 | 4627 | MYH9 |
| NAGK | 55577 | NAGK |
| NCAN | 1463 | NCAN |
| NDRG1 | 10397 | NDRG1 |
| NDST1 | 3340 | NDST1 |
| NDST2 | 8509 | NDST2 |
| NEDD4L | 23327 | NEDD4L |
| NFIL3 | 4783 | NFIL3 |
| NR3C1 | 2908 | NR3C1 |
| P4HA1 | 5033 | P4HA1 |
| P4HA2 | 8974 | P4HA2 |
| PAM | 5066 | PAM |
| PCK1 | 5105 | PCK1 |
| PDGFB | 5155 | PDGFB |
| PDK1 | 5163 | PDK1 |
| PDK3 | 5165 | PDK3 |
| PFKFB3 | 5209 | PFKFB3 |
| PFKL | 5211 | PFKL |
| PFKP | 5214 | PFKP |
| PGAM2 | 5224 | PGAM2 |
| PGF | 5228 | PGF |
| PGK1 | 5230 | PGK1 |
| PGM1 | 5236 | PGM1 |
| PGM2 | 55276 | PGM2 |
| PHKG1 | 5260 | PHKG1 |
| PIM1 | 5292 | PIM1 |
| PKLR | 5313 | PKLR |
| PKP1 | 5317 | PKP1 |
| PLAC8 | 51316 | PLAC8 |
| PLAUR | 5329 | PLAUR |
| PLIN2 | 123 | PLIN2 |
| PNRC1 | 10957 | PNRC1 |
| PPARGC1A | 10891 | PPARGC1A |
| PPFIA4 | 8497 | PPFIA4 |
| PPP1R15A | 23645 | PPP1R15A |
| PPP1R3C | 5507 | PPP1R3C |
| PRDX5 | 25824 | PRDX5 |
| PRKCA | 5578 | PRKCA |
| PRKCDBP | 112464 | CAVIN3 |
| PTRF | 284119 | CAVIN1 |
| PYGM | 5837 | PYGM |
| RBPJ | 3516 | RBPJ |
| RORA | 6095 | RORA |
| RRAGD | 58528 | RRAGD |
| S100A4 | 6275 | S100A4 |
| SAP30 | 8819 | SAP30 |
| SCARB1 | 949 | SCARB1 |
| SDC2 | 6383 | SDC2 |
| SDC3 | 9672 | SDC3 |
| SDC4 | 6385 | SDC4 |
| SELENBP1 | 8991 | SELENBP1 |
| SERPINE1 | 5054 | SERPINE1 |
| SIAH2 | 6478 | SIAH2 |
| SLC25A1 | 6576 | SLC25A1 |
| SLC2A1 | 6513 | SLC2A1 |
| SLC2A3 | 6515 | SLC2A3 |
| SLC2A5 | 6518 | SLC2A5 |
| SLC37A4 | 2542 | SLC37A4 |
| SLC6A6 | 6533 | SLC6A6 |
| SRPX | 8406 | SRPX |
| STBD1 | 8987 | STBD1 |
| STC1 | 6781 | STC1 |
| STC2 | 8614 | STC2 |
| SULT2B1 | 6820 | SULT2B1 |
| TES | 26136 | TES |
| TGFB3 | 7043 | TGFB3 |
| TGFBI | 7045 | TGFBI |
| TGM2 | 7052 | TGM2 |
| TIPARP | 25976 | TIPARP |
| TKTL1 | 8277 | TKTL1 |
| TMEM45A | 55076 | TMEM45A |
| TNFAIP3 | 7128 | TNFAIP3 |
| TPBG | 7162 | TPBG |
| TPD52 | 7163 | TPD52 |
| TPI1 | 7167 | TPI1 |
| TPST2 | 8459 | TPST2 |
| UGP2 | 7360 | UGP2 |
| VEGFA | 7422 | VEGFA |
| VHL | 7428 | VHL |
| VLDLR | 7436 | VLDLR |
| WISP2 | 8839 | CCN5 |
| WSB1 | 26118 | WSB1 |
| XPNPEP1 | 7511 | XPNPEP1 |
| ZFP36 | 7538 | ZFP36 |
| ZNF292 | 23036 | ZNF292 |
|  |  |  |
