# Supplementary material for: Identification of Immune and Hypoxia Risk Classifier to Estimate Immune Microenvironment and Prognosis in Cervical Cancer
Source: J Oncol. 2022 Oct 17;2022:6906380. doi: 10.1155/2022/6906380 (PMC9593224; doi:10.1155/2022/6906380)
Supplement: Supplementary 2 — Supplementary Table 2: potential function of the target miRNAs. [file 6906380.f2.zip › Supplementary Table 2.docx]

Supplementary Table 2: Potential function of the target miRNAs.

| Category | Term | Count | Percent | Fold | P-value | FDR |
| --- | --- | --- | --- | --- | --- | --- |
| Function | Apoptosis | 24 | 0.226415 | 8.283019 | 4.01E-18 | 2.35E-15 |
| Function | Immune Response | 24 | 0.26087 | 9.543478 | 1.01E-19 | 8.90E-17 |
| Function | Aging | 20 | 0.31746 | 11.61376 | 4.02E-18 | 1.76E-15 |
| Function | Inflammation | 20 | 0.178571 | 6.532738 | 8.86E-13 | 1.30E-10 |
| Function | Regulation of Stem Cell | 20 | 0.253165 | 9.261603 | 6.13E-16 | 1.54E-13 |
| Function | Cell Death | 18 | 0.230769 | 8.442308 | 1.58E-13 | 3.08E-11 |
| Function | Hormone-mediated Signaling Pathway | 18 | 0.310345 | 11.35345 | 4.68E-16 | 1.37E-13 |
| Function | Cell Cycle | 17 | 0.204819 | 7.492972 | 7.42E-12 | 8.14E-10 |
| Function | Cell Proliferation | 16 | 0.2 | 7.316667 | 5.36E-11 | 5.23E-09 |
