# Supplementary material for: Identification of Immune and Hypoxia Risk Classifier to Estimate Immune Microenvironment and Prognosis in Cervical Cancer
Source: J Oncol. 2022 Oct 17;2022:6906380. doi: 10.1155/2022/6906380 (PMC9593224; doi:10.1155/2022/6906380)
Supplement: Supplementary 2 — Supplementary Table 2: potential function of the target miRNAs. [file 6906380.f2.zip › Supplementary Table 2.pdf]

Supplementary Table 2: Potential function of the target miRNAs.

Category	Term	Count	Percent	Fold	P-value	FDR
Function	Apoptosis	24	0.226415	8.283019	4.01E-18	2.35E-15
Function	Immune Response	24	0.26087	9.543478	1.01E-19	8.90E-17
Function	Aging	20	0.31746	11.61376	4.02E-18	1.76E-15
Function	Inflammation	20	0.178571	6.532738	8.86E-13	1.30E-10
Function	Regulation of Stem Cell	20	0.253165	9.261603	6.13E-16	1.54E-13
Function	Cell Death	18	0.230769	8.442308	1.58E-13	3.08E-11
Function	Hormone-mediated Signaling Pathway	18	0.310345	11.35345	4.68E-16	1.37E-13
Function	Cell Cycle	17	0.204819	7.492972	7.42E-12	8.14E-10
Function	Cell Proliferation	16	0.2	7.316667	5.36E-11	5.23E-09
